# Oral bacteria in infective endocarditis requiring surgery: a retrospective analysis of 134 patients

**DOI:** 10.1007/s00784-022-04465-2

**Published:** 2022-03-22

**Authors:** Herbert Deppe, Julia Reitberger, Alexandra V. Behr, Keti Vitanova, Rüdiger Lange, Nina Wantia, Stefan Wagenpfeil, Anton Sculean, Lucas M. Ritschl

**Affiliations:** 1 Department of Oral and Maxillofacial Surgery, Technical University of Munich, Hospital “Rechts Der Isar”, Ismaninger Straße 22, 81675 Munich, Germany; 2 Department of Cardiac Surgery, German Heart Center Munich, Technical University of Munich, Lazarettstraße 36, 80636 Munich, Germany; 3 Department of Microbiology, Technical University of Munich, Hospital “Rechts Der Isar”, Ismaninger Straße 22, 81675 Munich, Germany; 4 Institute for Medical Biometry, Epidemiology and Medical Informatics, University of Saarland, Kirrbergerstraße Building 86, 66421 Homburg, Saar, Germany; 5 Department of Periodontology, University of Bern, Freiburgstrasse 7, 3010 Bern, Switzerland

**Keywords:** Infective endocarditis, Cardiac valve surgery, Periodontal bacteria, Dental treatment

## Abstract

**Objectives:**

It has been reported that bacteria associated with infective endocarditis originate from the oral cavity in 26–45% of cases. However, little is known on the counts and species of periodontal microbiota in infected heart valves. The aim of this study was to identify these aspects of periodontal microbiota in infective endocarditis and to potentially initiate a dental extraction concept for periodontally compromised teeth concerning patients requiring heart valve surgery.

**Materials and methods:**

The retrospective study group consisted of tissue samples from infected heart valves of 683 patients who had undergone heart valve surgery. Before patients had undergone cardiac surgery, the following laboratory tests confirmed the occurrence of endocarditis in all patients: blood cultures, echocardiography, electrocardiography, chest X-ray, and electrophoresis of the serum proteins. The specimens were aseptically obtained and deep frozen immediately following surgery. Microbiological diagnosis included proof of germs (dichotomous), species of germs, and source of germs (oral versus other).

**Results:**

Microbiota was detected in 134 (31.2%) out of 430 enrolled patients. Oral cavity was supposed to be the source in 10.4% of cases, whereas microbiota of the skin (57.5%) and gastrointestinal tract (GIT, 24.6%) were detected considerably more frequently. Moreover, periodontal bacteria belonged mostly to the *Streptococci* species and the yellow complex. None of the detected bacteria belonged to the red complex.

**Conclusion:**

Most frequently, the skin and GIT represented the site of origin of the microbiota. Nevertheless, the oral cavity represented the source of IE in up to 10%.

Consequently, it needs to be emphasized that a good level of oral hygiene is strongly recommended in all patients undergoing heart valve surgery in order to reduce the bacterial load in the oral cavity, thereby minimizing the hematogenous spread of oral microbiota. The prerequisites for conservative dental treatment versus radical tooth extraction must always be based on the patient’s cooperation, and the clinical intraoral status on a sense of proportion in view of the overall clinical situation due to the underlying cardiac disease.

**Clinical relevance:**

The oral cavity is a source of oral microbiota on infected heart valves. Patients requiring heart valve surgery should always undergo a critical evaluation of dental treatment affecting periodontally compromised teeth, favoring a systematic, conservative-leaning recall.

## Introduction

Over the past decade, cardiac valve surgery has become increasingly common in Germany. By the year 2019, annual interventions had increased to more than 36,650 according to the German Society for Thoracic, Cardiac, and Vascular Surgery. At least one reason for this observation could be the increased incidence of aortic stenosis in the elderly and the increase in elderly citizens overall. However, both compromised natural and artificial heart valves may be colonized by microbiota as a result of bacteremia, which may cause infective endocarditis (IE), especially as left-sided native valve endocarditis [7]. The incidence of IE in Europe is reported as 3–10 cases per 100,000 inhabitants annually [5] and in Canada as 1.5–4.95 [13]. This serious disease is characterized by elevated morbidity and mortality rates, with the latter reaching 30% [16], but varying substantially between studies from 11 to 64% [4, 7, 9, 12, 20].

It was reported that the respective bacteria originate from the oral cavity and teeth (26%), gastrointestinal tract (12.5%), drug abuse (5%), skin (5%), urinary tract (4%), iatrogenic (9%), and other origin (5.5%), while many are still undetermined (33%) [30]. Other authors found that oral bacteria are implicated in 35–45% of cases [28]. Methicillin-resistant *Staphylococcus aureus* species, diverse streptococcal strains (particularly *Streptococcus viridans*), and *Enterococcus faecalis* are the most frequent infective pathogens associated with native-valve endocarditis, and also in the late form of this disease following valve replacement [30]. In turn, the most frequent bacterial species present in gangrenous pulp is *Streptococcus viridans*.

Accordingly, it may be assumed that the coincidence of microbiota typical for dental pulp infections and that for IE appears not haphazardly. However, it was also emphasized that the amounts of periodontitis-related bacteria including *T. denticola* were negligible in infected heart valves, and therefore, it was speculated that *S. mutans* is a causative agent for cardiovascular disease [15]. Accordingly, evidence on the role of periodontopathogenic germs in infected heart valves is still a matter of ongoing debate.

Therefore, the aim of the present study was to analyze bacteria from infected heart valves and to identify the percentage of oral microbiota and periodontopathogenic bacteria. It was the hypothesis of this study that oral bacteria and periodontopathogenic bacteria do not significantly contribute to IE, which might strengthen concepts of preserving periodontally compromised teeth before heart valve replacement.

## Materials and methods

### Patients

The study was performed in accordance with the ethical standards of the Declaration of Helsinki and approved by the institutional ethics board of the Technical University of Munich, Klinikum rechts der Isar (approval no. 232/16S).

The study group consisted of tissue samples obtained from infected heart valves of 683 patients who had undergone heart valve surgery at the Department of Cardiovascular Surgery, German Heart Center Munich, between May 2009 and May 2020. All of the heart valve specimens had been excised during a valve replacement procedure, following diagnosis of aortic regurgitation, mitral regurgitation, or tricuspid regurgitation. These specimens were aseptically obtained and deep frozen immediately following cardiac surgery and analyzed in the Department of Microbiology of the Technical University of Munich. Before patients had undergone cardiac surgery, the following laboratory tests confirmed the occurrence of endocarditis in all patients: blood cultures, echocardiography, electrocardiography, chest X-ray, and electrophoresis of the serum proteins. There has also been regarded to the Duke criteria [8]. Inclusion criteria were focused on the microbiological results after requiring surgery.

All medical information was extracted from the IE patients’ medical charts. Accordingly, medical histories of the patients concerning recent or recurrent IE and coexisting acquired heart disease were evaluated. Standardized forms allowed the documentation of age, gender, type of prosthesis (bio-prostheses, mechanical valve), and microbiological diagnosis. The latter included proof of germs (yes/no), species of germs, and source of germs (oral versus other).

### Microbiology

During surgical interventions, heart valve tissue samples were taken from clinically infected sites, harvested in Port-A-Cul™ (PAC) transport system tubes (BD Diagnostics; Heidelberg, Germany), and immediately transferred to the Department of Microbiology. In the laboratory, samples were directly cultured in brain–heart infusion (BHI) broth (bioMerieux; Nürtingen, Germany) and in thioglycolic medium (Oxoid Deutschland GmbH; Wesel, Germany), enriched with hemin (Sigma) and menadione (Merck; Darmstadt, Germany). Tissue samples were shredded using a sterilized mortar. For culture, samples were conducted to Columbia blood agar plate, chocolate agar plate, and Schaedler agar (anaerobic growth). The agar plates were incubated for 48 h at 36 °C. Without growth of bacteria after 48 h, the cultures were subsequently incubated for up to 14 days at a temperature of 36 °C. Visual controls from the BHI broth and the thioglycolic broth to blood disks and agar were performed regularly, and in the case of observed growth on the liquid medium, a passage was conducted to two agar plates (Columbia agar and Schaedler agar; BD Diagnostics; Heidelberg, Germany). Representative bacterial colonies growing in aerobic or anaerobic conditions on solid medium were passed in order to obtain pure bacterial cultures and were subsequently identified using biochemical tests with use of the systems Vitek 2 (bioMerieux) and in most cases matrix-assisted laser desorption/ionization/ time of flight (MALDI-TOF) analysis (Bruker Daltonics GmbH; Bremen, Germany). Antibiotic susceptibility testing was performed according to EUCAST regulations using the VITEK 2 system or standardized disk diffusion or MIC determination by agar dilution. The Microbiological Laboratory is certified according to Deutsche Akkreditierungsstelle ML-14063-01-00 since 2009.

To allow comparison with the literature, identified microbiota were assigned to one of seven groups described by Corvec et al. [1]: coagulase-negative staphylococci, *Staphylococcus aureus*, gram-negative rods (aerobic), enterococci, anaerobes (facultative and obligate), streptococci, and others.

Microbiological analysis was also focused on the identification of the following periodontopathogenetic bacteria and complexes (red, orange, yellow, green, and purple) defined by Socransky et al. [27]. The red complex includes *Bacteroides forsythus*, *Porphyromonas gingivalis*, and *Treponema denticola*. The orange complex consists of *Fusobacterium nucleatum/periodonticum* subspecies, *Prevotella intermedia*, *Prevotella nigrescens* and *Peptostreptococcus micros*, *Eubacterium nodatum*, *Campylobacter rectus*, *Campylobacter showae*, *Streptococcus constellatus*, and *Campylobacter gracilis*. The yellow complex consists of *Streptococcus sanguis*, *S. oralis*, *S. mitis*, *S. gordonii*, and *S. intermedius*. The green complex includes *Capnocytophaga* species, *Campylobacter concisus*, *Eikenella corrodens*, and *Actinobacillus actinomycetemcomitans serotype a*. The purple complex consists of *Veillonella parvula* and *Actinomyces odontolyticus*. *A. actinomycetemcomitans serotype b*, *Selenomonas noxia*, and *Actinomyces naeslundii genospecies 2* (*A. viscosus*) were outliers with little relation to each other and the five major complexes.

### Statistical analysis

The data were analyzed by means of the Statistical Package for Social Sciences program (SPSS 19.0, Chicago III, USA). Data of the microbiological parameters are presented as means ± standard deviation for quantitative and relative as well as absolute frequencies for qualitative data. Statistical testing comparing mean of two independent samples for quantitative variables was performed using Student’s *t* test. A two-sided *P*-value less than 0.05 was considered to indicate statistical significance.

## Results

### Patients

**Fig. 1:**
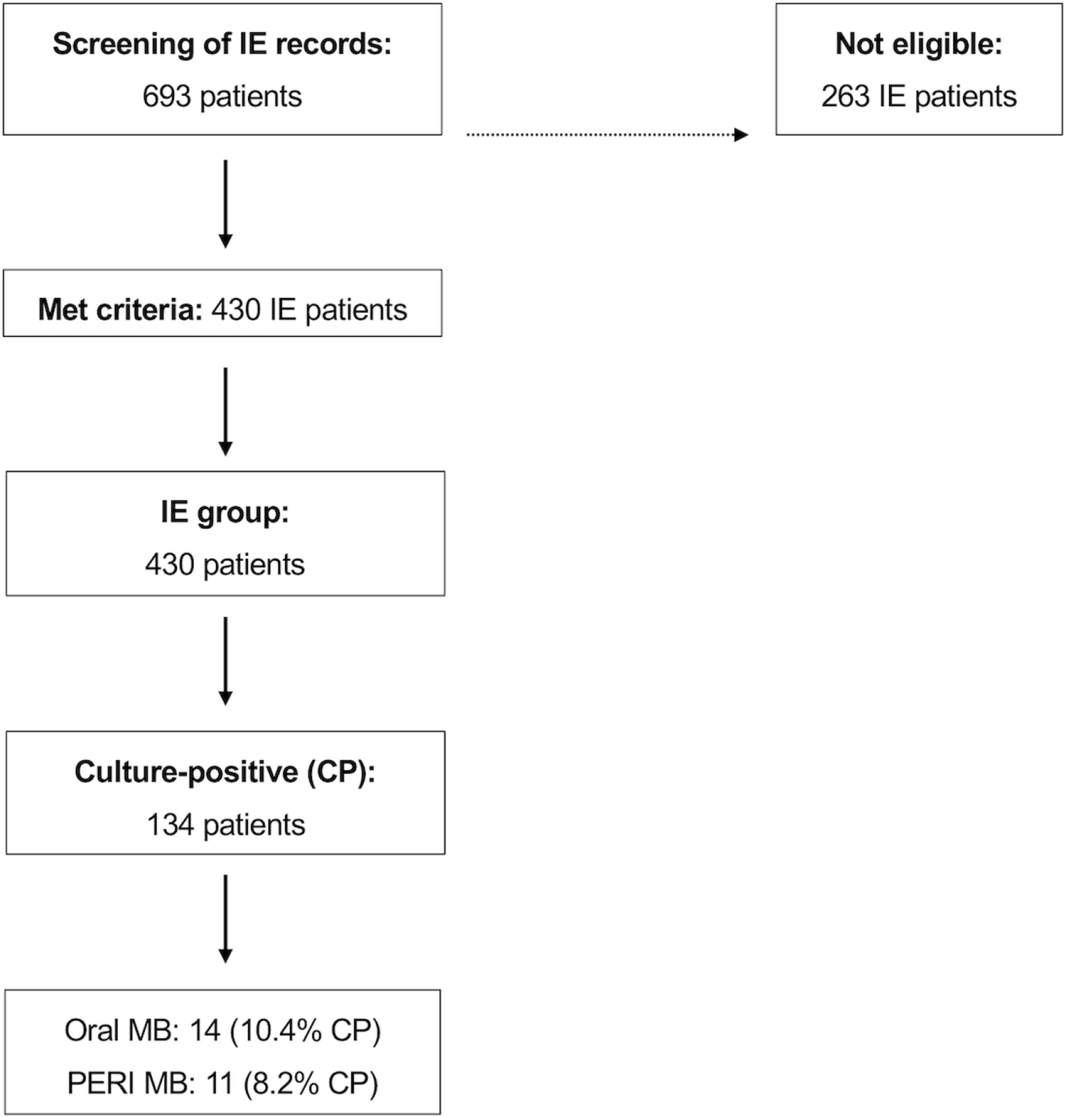
Flow chart of patients and infections. (CP culture positive; oral MB oral microbiota; PAR MB periodontal microbiota; IE infective endocarditis)

From the 693 IE patients whose records had been screened, a total of 430 fulfilled the inclusion criteria (Fig. 1). Of these eligible patients, a total of 134 heart valve specimens from 104 males (77.6%) and 30 females (22.4%) were culture-positive and allowed further evaluation (average age 61.8 ± 14.7 years; range 28–86 years) (Table 1). Similarly, in individuals with culture-negative isolates, the mean age of patients in the IE patients (63.2 ± 13.8 years) differed statistically significantly (*p* < 0.01) from that of culture-negative controls (65.9 ± 14.2 years).

**Table 1:** Characteristics of the enrolled patients with culture-positive infective endocarditis (IE)

Variable	Study participants (*n* = 430)
Culture-positive isolates	134 (31.2%)
Culture-negative isolates	296 (68.8%)
Gender of patients with culture-positive IE (male/female; *n*, %)	104 (77.6%) /30 (22.4%)
Age of patients with culture-positive IE (years; mean ± SD; range)	61.8 ± 14.7 (28–86)
Age of patients with culture-negative IE (years; mean ± SD; range)	63.2 ± 13.8 (25–91)
Infection with oral bacteria	14 (10.4%)
Infection with periodontal bacteria	11 (8.2%)

**Fig. 2:**
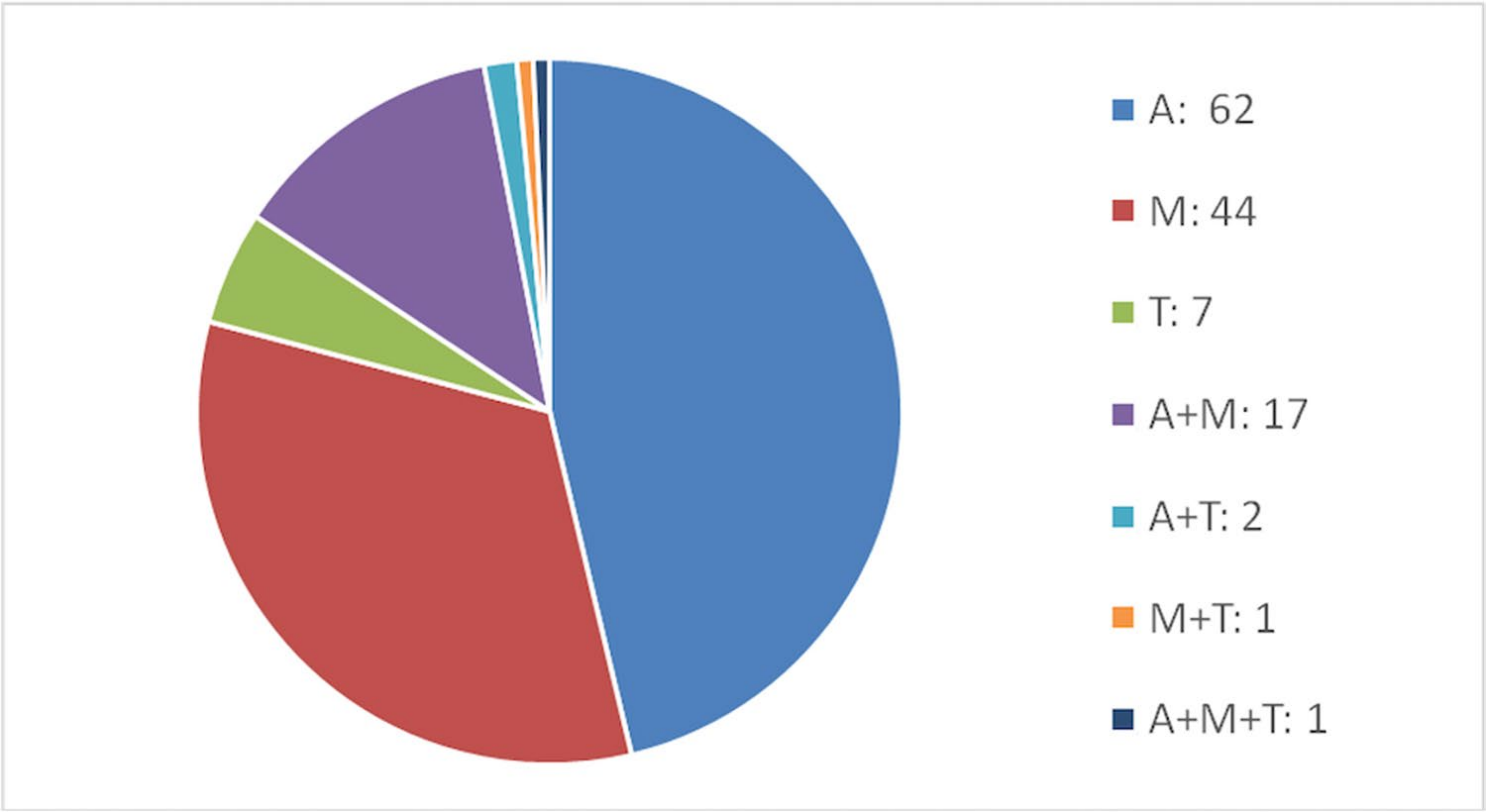
Counts and distribution of infected valves (*n* = 134). (A aortic valve; M mitral valve; T tricuspid valve and combinations of these)

The distribution of valve involvement is illustrated in Fig. 2 and shows the vast majority of all 134 IE cases at the aortic valves (*n* = 62) and mitral valves (*n* = 44). Tricuspid valves and combinations of valves showed significantly lower counts. With respect to the nature of infected valves, it was seen that 86 cases were attributable to native valves, 32 cases to biological valve prostheses, and five cases to mechanical valve prostheses.

### Microbiology

The microbiological detection rate was determined in the IE patients (Table 1). Microbiota had been detectable in 134 (31.2%) out of the 430 patients from the study participants.

Table 2 shows also the counts and percentages of microbiota which contributed to microbial infections in culture-positive isolates, according to the seven groups of microbiota [1]. The seven groups of microbiota did not contribute comparably to microbial infections in IE. Significant differences were seen in coagulase-negative staphylococci (CNS), in *Staphylococcus aureus*, enterococci, and anaerobes. In contrast, statistical analyses failed to detect significance for the streptococci species.

**Table 2:** Identified microbiota of culture-positive heart valves according to the seven groups of microbiota described by Corvec et al.

	Study participants % (*n* = 126)
*Coagulase-negative staphylococci*	21.4 (27)
Staphylococcus aureus	25.4 (32)
Gram negative rods (aerobic)	8.7 (11)
Enterococci	20.6 (26)
Anaerobes (facultative and obligate)	3.2 (4)
Streptococci	14.3 (18)
Others	6.3 (8)

With respect to microbiota species, a wide variety of species were found in the IE patient heart valves (Table 3). Most of them were supposed to have originated from the skin (57.5%; *Staphylococcus aureus*, MRSA, *Staphylococcus epidermidis*, *Staphylococcus lugdunensis*, *Staphylococcus hominis*, *Staphylococcus capitis*, *Staphylococcus haemolyticus*, *Micrococcus luteus*, coagulase-negative staphylococci, *Cutibacterium acnes*, *Cutibacterium granulosum*, *Propionibacterium acnes*). Other sources of microbiota were the gastrointestinal tract (24.6%; *Enterococcus faecalis*, *Streptococcus gallolyticus*, *E. coli*, *Klebsiella pneumoniae*, *Proteus mirabilis*), the environment (0.7%; *Serratia marcescens*), and the genitourinary system (0.7%; Group B streptococci). The oral cavity was supposed to be the source of the following microbiota (10.4%): *Streptococcus gordonii* (2/14), *Streptococcus mitis* (5/14), *Streptococcus sanguis* (3/14), *Streptococcus oralis* (1/14), *Granulicatella adiacens* (1/14), and *Abiotrophia defective* (2/14). Moreover, three fungal species were detected: *Candida albicans* (2/14), *Candida glabrata* (2/14), and *Candida dubliniensis* (1/14).

**Table 3:** Origin of identified bacteria (counts of patients per group of bacteria; multiple categories possible)

Assumed origin of bacteria	Study participants % (*n* = 134)
Skin	57.5 (77)
Gastro-Intestinal Tract	24.6 (33)
Environment	0.7 (1)
Genitourinary system	0.7 (1)
Oral Cavity	10.4 (14)

### Oral microbiota

Among the 134 culture-positive IE patients, oral microbiota were identified in a total of 14 IE patients (10.4%) (Fig. 1). Thus, oral microbiota displayed a presence in IE patients.

The distribution of periodontal bacteria species is presented in Table 4. The vast majority of periodontal bacteria were attributable to the streptococci species. Statistical analysis failed to indicate significance (*p* = 0.053). With respect to the 20 most important periodontal pathogenic bacteria [11], a total of seven could be detected in this study (Table 4). Among all 134 culture-positive patients, periodontal microbiota was identified in 11 patients (8.2%) from the study participants (Fig. 1).

**Table 4:** Identified periodontal species of bacteria (*n* = counts of patients per species; multiple categories if more than one species was found in one patient)

	Study participants (*n* = 11/14)	*p*-value
Streptococcus spp.	*S. constellatus*: 0 *S. gordonii*: 2 *S. intermedius*: 0 *S. mitis*: 5 *S. oralis*: 1 *S. sanguinis*: 3 Ʃ 11	0.053
Peptostreptococcus spp.	*P. micra*: 0 Ʃ 0	
Actinomyces spp.	*A. odontolyticus*: 0 Ʃ 0	

**Fig. 3:**
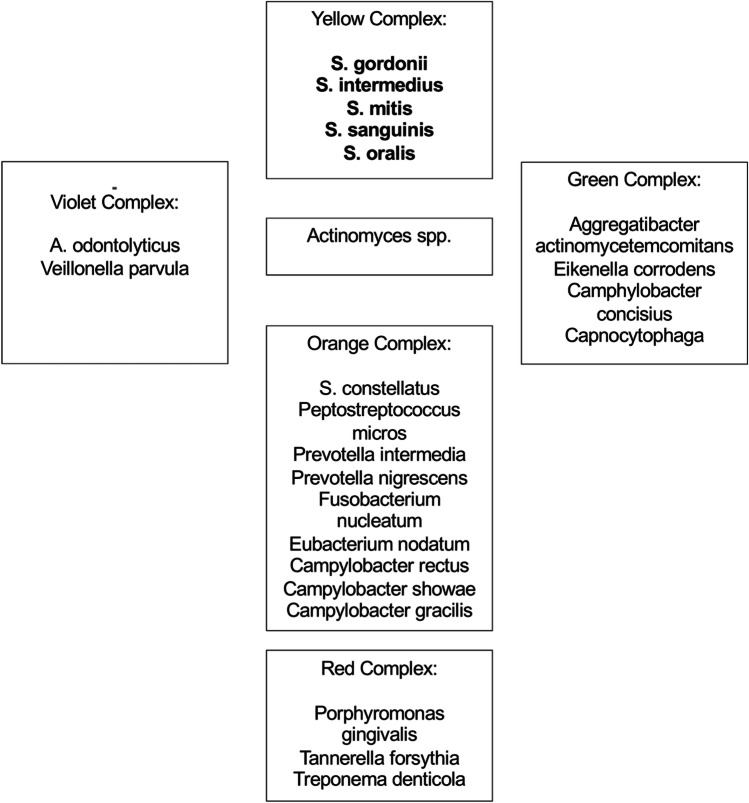
Periodontal bacteria in the IE patients with respect to dental region according to [14, 27] (bold — identified bacteria)

All periodontal pathogenic bacteria of the study participants belonged to the yellow complex. None of the microbiota belonging to the red complex could be detected (Fig. 3).

## Discussion

People with valvular disease undergoing cardiac valve replacement are at increased risk of developing IE postoperatively [3]; community-associated IE is the most common form and often linked to oral bacteria [3, 11]. However, from the available evidence, it is unclear whether postoperative outcomes differ in patients receiving dental treatment before cardiac valve surgery compared with outcomes in those who do not [11]. At present, it is also unclear whether or not periodontopathogenic bacteria significantly contribute to IE. Nevertheless, since 1955, expert committees have made several recommendations for antimicrobial prophylaxis for the prevention of bacterial endocarditis following dental procedures [23]. In the light of the significant morbidity associated with IE and consecutively the enormous economic burden per infection episode, preventing such infections in patients undergoing dental procedures is highly desirable. However, it was recently shown that dentists reported uncertainty about the appropriate use of antibiotic prophylaxis as defined in the IE guidelines [11]. Therefore, there is a persisting need for clinical studies on the role of oral and especially periodontal bacteria in IE to find out whether or not patients should undergo dental treatment to what extent before heart valve surgery. Accordingly, in this study, data on IE-related microbial diagnosis were evaluated on the role of oral microbiota to set up instructions for dental extraction concepts concerning periodontally compromised teeth in patients requiring cardiac valve surgery.

Smith et al. [26] evaluated perioperative major adverse events in patients who underwent dental extraction prior to cardiovascular surgery. In their study, the mean age of patients was 60 ± 15 years (range 18–92 years), which is close to the demographic data of culture-positive IE patients in this study (63.2 ± 13.8, range 25–91 years). Comparable results were also found by Oliveira et al. [19] and Rao et al. [21] reporting that more male than female individuals underwent cardiac valve surgery (54.8% versus 45.2% and 58.9% versus 41.8%, respectively). Accordingly, the present study population is comparable to similar study population groups in terms of gender and mean age and may be considered as representative.

In this study, microbiological detection rates were evaluated from patients with culture-positive IE, which are also in agreement with the literature. It was emphasized that the etiology of IE cannot be clarified in up to 31% of cases, even though diagnostic methods have been improved in the past [22]. In this context, valve sequencing was considered to be significantly more sensitive than valve culture in identifying the causative pathogen (90% versus 31%, *p* < 0.001) and yielded fewer false positive results [25]. In contrast, in another study, the species determined by the blood culture technique were not always identified by the molecular methods [18]. Although the reasons for lower detection rates in our study remain unclear, another potential explanation may be that many patients had undergone antibiotic therapy before they were referred to this center. It has been shown that the administration of systemic antibiotics before microbiological diagnosis may substantially affect the results of such analysis [14]. Another explanation might be the fact that species may have incidentally disseminated into the bloodstream. Thus, it was concluded that the interpretation of such results should be undertaken carefully in clinical situations [18].

The literature has pointed out that microbial infections may indicate the hematogenous spreading of oral bacteria [10]. In this study, the seven groups of microbiota according to Corvec et al. [1] did not contribute comparably to mono-microbial infections in IE patients. Statistically significantly high percentages were seen in IE patients, for *Staphylococcus aureus* and for enterococci, whereas CNS and anaerobes were represented in lower percentages in IE patients. However, statistical analyses failed to detect significance for streptococci species. These findings are, in part, in accordance with the literature [2, 6]. Delahaye and coworkers [2] have stated that the skin was the most frequent (40%) portal of entry for microorganisms (*Staphylococcus aureus*) in IE patients while the second most frequent was the oral cavity (29%). In contrast, Westphal and coworkers stated that typical entry points of microbiota in IE patients are the oral cavity and teeth (26%), the gastrointestinal tract (12.5%), drug abuse (5%), the skin (5%), the urinary tract (4%), iatrogenic (9%), other (5.5%), and undetermined (33%) [29]. The reasons for the different distributions are unknown. It may be speculated that the different study groups (including drug abuse or not) may be causative. In any case, in the present study, streptococci species were seen in the IE patients whose source was most probably the oral cavity (10.4%). This finding supports, again, the notion that oral bacteria spread hematogenously, which has been proven most recently for *Streptococcus mutans* in an animal study in rats [17]. *Streptococcus mutans* is known to be one of the pathogens that cause IE, though it is primarily a major cariogenic pathogen that is a normal inhabitant of the oral cavity in most individuals [15]. Therefore, early treatment of dental caries and good oral hygiene should also be considered as cardio-preventive measures.

For many years, studies have been developed with an aim to investigate the possible connection between periodontal disease and cardiovascular diseases such as IE [19]. The presence of oral bacteria in the bloodstream (bacteremia) is probably one of the initiators of biological events that justify this association [15]. In a more recent study on infected heart valves, only 10.6% of the specimens were not infected with cariogenic or periodontopathic bacteria [19]. In detail, it was reported that the most frequently detected periodontal microorganisms in heart valve samples were *Prevotella intermedia* (*P. intermedia*) (19.1%), *Porphyromonas gingivalis* (*P. gingivalis*) (4.2%), and *Treponema denticola* (*T. denticola*) (2.1%). Among these bacteria, *P. intermedia* was present in statistically significantly higher numbers in the valve tissue compared to *P. gingivalis* (*p* = 0.025) and *T. denticola* (*p* = 0.007) [19]. These three bacteria species are part of the orange and red complexes according to the periodontal complex theory [27]. Also in our study, several periodontal micro-organisms were detected such as *S. gordonii*, *S. intermedius*, *S. mitis*, *S. sanguinis*, and *S. oralis*, which belong to the yellow complex [27]. No bacteria of the orange or red complexes could be detected (Table 3). One potential explanation for this difference is due to the fact that Oliveira and coworkers excluded edentulous patients (44.0%), whereas a greater number of patients with periodontal pockets larger than four millimeters (43.4%) and dental calculus (34.7%) were included [19]. Due to the retrospective character of the present study, dental findings were not assessed and, therefore, percentages of edentulous patients or individuals with deeper pockets are unknown. Accordingly, the difference in the aforementioned bacterial complexes must be interpreted with caution. Nevertheless, the lack of dental findings in this study seems not to hamper the significance of the results. In a recent study, it was pointed out that there was no statistically significant difference between the frequency of oral bacteria in the heart valves regarding the dental condition (dentate or edentulous) (*p* = 0.504), anatomical location (aortic or mitral) (*p* = 0.596), and clinical diagnosis (stenosis, insufficiency, or both) (*p* = 0.256) [19]. It therefore seems that the present results may be considered as reliable despite the fact that dental status was not assessed due to the retrospective character of the study. Nevertheless, it can be assumed that among the 134 culture-positive isolates of the IE, also patients with deeper pockets were included. All five periodontal bacterial species identified in the IE patients belonged to the yellow complex, which plays a subordinate role in the etiology of periodontitis and is also found in healthy oral cavities. Accordingly, it may be assumed that also simple and moderate forms of periodontitis and even bacteria of the healthy oral cavity may contribute to IE. Therefore, it is strongly recommended that patients should optimize their oral hygiene before heart valve replacement, including twice-daily teeth brushing, daily application of dental floss or interdental brush, and professional dental cleaning, as a minimum requirement. To achieve this, individually adapted techniques and aids should be recommended by the treating dentist. Patients should learn the correct use of these aids, if necessary with professional support and exercises, and the success of home oral hygiene measures (tooth brushing and interdental hygiene) should be checked in the short term. In this way, the bacterial load in the oral cavity should be reduced to minimize the hematogenous spread of periodontal microbiota. The direct influence of oral hygiene on cardiac diseases should be communicated in more detail to overcome uncertainty about the appropriate use of antibiotic prophylaxis as defined by IE guidelines [11].

### Limitations

When interpreting the results, limitations of the study need to be considered. First, as in all retrospective studies, there is a potential for variability in reports of clinical data provided by treating clinicians. The authors attempted to minimize bias through a priori definitions and data collection by a single researcher. Second, patients were recruited from an inpatient setting of a single university hospital. Therefore, the patients might not be representative of the entire population. Third, records did not comprise dental findings, and thus, it was impossible to analyze the periodontal health status and its impact on the rate of heart valve infections. Lastly, among biofilm analysis, as an integral precondition in the pathogenesis of caries, other parameters such as structure of dental hard tissues, environmental factors, immunocompetency, and genetic predisposition as well as interactions between components such as salivary flow and composition were not recorded and analyzed. For more fundamental and evidence-based recommendations of future studies, a larger study sample with a modern study design and more advanced investigation techniques in a prospective setting is necessary. Rodriguez-Garcia et al. described the impact of 16SrDNA tissue PCR for diagnostic complementation in IE patients with negative blood cultures. Such modern molecular diagnostics are important to obtain better information for the diagnosis of IE and to create preventive therapeutic treatment tools, including dental treatments [24].

## Conclusion

Most frequently, the skin and GIT represented the site of origin of the microbiota. Nevertheless, the oral cavity represented the source of IE in up to 10% with microbiota from the yellow complex, which plays a subordinate role in the etiology of periodontitis. Consequently, it needs to be emphasized that a good level of oral hygiene is strongly recommended in all patients undergoing heart valve surgery in order to reduce the bacterial load in the oral cavity, thereby minimizing the hematogenous spread of periopathogenic microbiota. The prerequisites for conservative dental treatment versus radical tooth extraction must always be based on the patient’s cooperation and the clinical intraoral status with a sense of proportion in view of the overall clinical situation due to the underlying cardiac disease.
